# Hemostatic Biomarkers and Volumetry Help to Identify High-Risk Abdominal Aortic Aneurysms

**DOI:** 10.3390/life12060823

**Published:** 2022-05-31

**Authors:** Sebastian Fernandez-Alonso, Esther Martinez-Aguilar, Susana Ravassa, Josune Orbe, Jose A. Paramo, Leopoldo Fernandez-Alonso, Carmen Roncal

**Affiliations:** 1 Departamento de Angiología y Cirugía Vascular, Hospital Universitario de Navarra, 31008 Pamplona, Spain; s.fernandez.alonso@navarra.es (S.F.-A.); esther.martinez.aguilar@navarra.es (E.M.-A.); leopoldo.fernandez.alonso@navarra.es (L.F.-A.); 2 Instituto de Investigación Sanitaria de Navarra—IdiSNA, 31008 Pamplona, Spain; sravassa@unav.es (S.R.); josuneor@unav.es (J.O.); japaramo@unav.es (J.A.P.); 3 Laboratory of Heart Failure, Program of Cardiovascular Diseases, Cima Universidad de Navarra, 31008 Pamplona, Spain; 4 Centro de Investigación Biomédica en Red Cardiovascular—CIBERCV, 28029 Madrid, Spain; 5 Laboratory of Atherothrombosis, Program of Cardiovascular Diseases, Cima Universidad de Navarra, 31008 Pamplona, Spain; 6 Hematology Service, Clínica Universidad de Navarra, 31008 Pamplona, Spain

**Keywords:** abdominal aortic aneurysm, angiography, volumetry, thrombosis, hemostasia, surgery

## Abstract

Predicting the progression of small aneurysms is a main challenge in abdominal aortic aneurysm (AAA) management. The combination of circulating biomarkers and image techniques might provide an alternative for risk stratification. We evaluated the association of plasma TAT complexes (TAT) and D-dimer with AAA severity in 3 groups of patients: group 1, without AAA (*n* = 52), group 2, AAA 40–50 mm (*n* = 51) and group 3, AAA > 50 mm (*n* = 50). TAT (*p* < 0.001) and D-dimer (*p* < 0.001) were increased in patients with AAA (groups 2 and 3) vs. group 1. To assess the association between baseline TAT and D-dimer concentrations, and AAA growth, aortic diameter and volume (volumetry) were measured by computed tomography angiography (CTA) in group 2 at recruitment (baseline) and 1-year after inclusion. Baseline D-dimer and TAT levels were associated with AAA diameter and volume variations at 1-year independently of confounding factors (*p* ≤ 0.044). Additionally, surgery incidence, recorded during a 4-year follow-up in group 2, was associated with larger aneurysms, assessed by aortic diameter and volumetry (*p* ≤ 0.036), and with elevated TAT levels (sub-hazard ratio 1.3, *p* ≤ 0.029), while no association was found for D-dimer. The combination of hemostatic parameters and image techniques might provide valuable tools to evaluate AAA growth and worse evolution.

## 1. Introduction

The abdominal aortic aneurysm (AAA) is a pathological arterial dilation that predisposes to its rupture, with an associated mortality greater than 70% [1]. Currently there is no pharmacological therapy that prevents the growth of AAA [2], so early diagnosis and evolution monitoring are essential to prevent its rupture.

Surgery is the only treatment for AAA. Its indication, either open or endovascular, is based on the maximum aortic diameter, this being the most widely used quantitative criterion for screening, follow-up or therapeutic indication [3]. The lack of consensus on maximum aortic diameter measurement protocol [3,4,5], and the possible use of other techniques for the three-dimensional assessment of aneurysms have open up the posibility of implementing other image methods for AAA evaluation. In this context, some authors have explored the usefulness of volumetry for AAA assessment in the follow-up of aneurysms treated endovascularly [6,7], although little is known about its utility to evaluate non-surgical aortic aneurysm [8,9]. Various authors defend that volumetry provides more information than aortic diameter since it fully evaluates the aneurysmal sac in a three-dimensional manner and can detect changes in the size of the aneurysm independently of the aortic diameter [10,11].

Without reaching rupture, the mere presence of an AAA is strongly associated with cardiovascular disease [12]. The United Kingdom Small Aneurysm Trial observed a cardiovascular mortality of 28% in a period of 8 years for patients with small AAA [13], being the risk of rupture higher as the size of the aneurysm increases [14]. In search of diagnosis and prognosis biomarkers for AAA patient stratification, and considering the the pathophysiological changes underlying AAA growth and intraluminal thrombus formation, closely related to deranged hemostasis, some authors have studied the possible role of hemostatic proteins in this context. As such, a recent meta-analysis suggests the existence of elevated levels of various coagulation markers such as fibrinogen, D-dimer or thrombin/antithrombin complexes (TAT) in patients with AAA and its association with cardiovascular mortality [12]. 

The current study pursued 4 objetives: (1) To explore the utility of the hemostatic markers, TAT complex and D-dimer, as bimomarkers of AAA by comparing their levels in atherosclerotic patients without AAA, also known to display a hipercoagulable state, and patients with AAA. (2) To determine, in a subgroup of patients with non-surgical AAA, aneurysm growth by computed tomography angiography (CTA) over a year, and assess the performance of the maximun aortic diameter and the volumetry for the early detection of AAA growth. (3) To evalute the correlation between TAT and D-dimer levels and AAA growth over a year. (4) To analyze the utility of image parameters, maximum aortic diameter and volume, and hemostatic markers to estimate surgery incidence in patients with non-surgical AAA during a mean follow-up of 4 years.

## 2. Materials and Methods

### 2.1. Study Design

Prospective study including 153 vascular patients enrolled at the outpatient service of the Vascular Surgery Department of the Complejo Hospitalario de Navarra (CHN) between June 2017 and June 2019. Patients were divided in: group 1 (*n* = 52), patients with atherosclerosis (coronary heart disease, cerebrovascular disease and/or peripheral artery disease) without AAA with aortic dimeters <30 mm; group 2 (*n* = 51), patients with AAA 40–50 mm; group 3, patients with AAA >50 mm eligible for surgery (*n* = 50). All patients in group 1 were studied by abdominal ultrasonography to ensure that the aortic size was <30 mm, the inclusion criteria for group 1.

Samples and data were provided by the Biobank of the University of Navarra and were processed following standard operating procedures approved by the Ethical and Scientific Committees. Exclusion criteria were: evidence of neoplastic disease, generalized or localized inflammatory disease (moderate or severe), severe chronic kidney disease (estimated glomerular filtration rate < 30 mL/min/1.73 m^2^) or hemodialysis, or recent thrombotic event (<3 months) including; acute coronary syndrome, stroke, venous thromboembolic disease, or acute artery ischemia. A thorough medical record was assembled for all patients including cases of previous myocardial infarction, non-ischemic cardiomyopathy, cerebrovascular disease and medication.

*Follow-up:* patients with AAA in group 2 were follow-up for a mean average of 4 years. Surgery, our primary outcome (*n* = 29, 57%), was recorded as a readout of the number of patients with AAA diameter growth reaching the surgical intervention limit. The critical diameter for surgical treatment was 55 mm. All-cause mortality was also recorded (*n* = 9, 18%) as a competing event for Cox regression analysis. 

The study was approved by the Institutional Review Board of the Complejo Hospitalario de Navarra (48/2014) according to the standards of the Declaration of Helsinki on medical research, and written informed consent was obtained from all patients who were enrolled in this study.

### 2.2. Laboratory Analysis and Hemostatic Parameters

Serum total cholesterol, HDL cholesterol, triglycerides and glucose were measured in fasting blood samples by standard laboratory techniques at recruitment. LDL cholesterol was estimated using the Friedewald equation. 

D-dimer and Thrombin/antithrombin (TAT) complexes were measured in citrated plasma samples obtained at patient recruitment. D-dimer levels were measured with the STA^®^ -Liatest^®^ D-Di Plus immunoassay (Stago). Thrombin/antithrombin complexes were determined with the Enzygnost^®^ TAT micro assay (Siemens Healthcare, Erlangen, Germany) following the manufacturer’s instructions with an inter- and intra-assay coefficient of variation of 6–9% and 4–6%, respectively, and a detection range of 2–60 µg/L. 

### 2.3. Image Analysis

**Figure 1:**
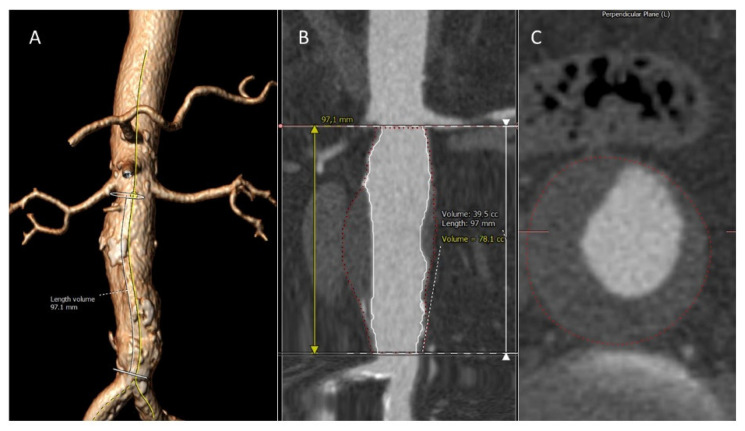
Volumetric image worksheet. (**A**) AAA 3D reconstruction with the centerline. (**B**) Results of the volumetry after the semi-automatic method. (**C**) CT slide showing the total volume area.

AAA size was determined by CTA. In group 2 an additional CTA was performed 1-year after recruitment, losing no patient to the follow-up. The obtained DICOM images were analyzed semi-automatically with the 3Mensio workstation (3Mensio Medical Imaging B.V. Bilthoven, The Netherlands) to obtain the maximum aortic diameter and the aneurysm total volume (Figure 1). 

The thrombus volume and the aortic lumen were determined in the same vascular segment obtained similarly in all patients. After performing an automatic centerline from the supra-renal aorta to both common iliac arteries, the first tomographic cut on which the renal plane was not visualized, and the last axial cut on which the aortic cone without separation between the two iliac arteries was visualized, were defined. To ensure that the studied segment was the same in each patient, the operator, a specialist in aortic surgical planning with the same software, analyzed all CTA scans (baseline and 12 months) at the same time, and was blinded to the order in which the CTAs were performed to avoid bias. Baseline and 1-year image analysis were performed by the same operator. 

### 2.4. Statistical Analysis

Continuous variables are expressed as mean ± SD or median (interquartile range), and categorical variables are expressed as percentages. Variables with non-gaussian distribution were logarithmically transformed. Differences among 3 groups were assessed by one-way ANOVA. Categorical variables were compared with the χ^2^ test or Fisher’s exact test. Mix model regression analysis was performed to determine the association of hemostatic parameters with AAA growth in group 2 adjusting for traditional risk factors of AAA (age, sex, smoking and dyslipidemia). Fine-Gray competing risk models were used to obtain sub-hazard ratios (SHR) and their 95% CI for surgery, considering all-cause death as a competing event after adjusting for the previously mentioned covariables. The proportional sub-hazard assumptions were verified using Schoenfeld’s residuals for each model. If violated, standard Cox or competitive risk regression analyses were extended including time varying covariates for each variable that did not satisfy this assumption. Results of this analysis are displayed graphically as Kaplan-Meier survival plots. Analyses were performed with SPSS version 25 and STATA version 13 (Stata Corp., College Station, TX, USA). All *p*-values are two-tailed, and statistical significance was set at *p* < 0.05.

## 3. Results

### 3.1. Baseline Characteristics and Hemostatic Parameters in Patients with AAA 

As shown in Table 1, the presence of traditional risk factors was similar in the 3 studied groups, except for the percentage of men and the frequency of smokers that progressively increased according to AAA size (*p* for trend <0.001). In addition, HDL levels decreased according to the size of AAA being lowest in group 3 (*p* for trend 0.002). Moreover, patients in group 3 were more often prescribed with antiplatelets (*p* < 0.009) and β-blockers (*p* = 0.049), whereas no differences were found for other drugs. Despite the high percentage of patients on statins, mean LDL-C cholesterol levels were above the recommended target concentration [5,15] in all studied groups. No differences were found in the coexisting CV diseases among the studied groups, except for peripheral artery disease (PAD), that was more predominant in group 1 (PAD: 54% group 1 vs 18% and 22% groups 2 y 3, respectively, *p* < 0.001). Regarding the levels of the coagulation biomarkers TAT and D-dimer, both progressively increased according to AAA size (*p* for trend < 0.001, Table 1). 

### 3.2. AAA Growth Assessment at 1-Year

To determine AAA diameter and volume progression, a second CTA was performed in the subgroup of patients with non-surgical AAA 1 year after recruitment (group 2, Figure 1). As shown in Table 2, aneurysm maximum diameter and volume, including total, thrombus and aortic lumen volumes, increased at 1-year versus baseline in group 2 (*p* ≤ 0.001) (Table 2). 

Then we calculated the differences between the measurements obtained with the second CTA scan and the first, and obtained the difference (δ) in AAA diameter, the δAAA volume, the δThrombus vol and the δaortic lumen vol. Interestingly, we found a positive association between the differences in the maximum aortic diameter (δAAA diameter) and AAA total volume (δAAA volume) (Pearson r = 0.37, *p* = 0.008), and between the δAAA volume and the δThrombus vol at 1-year vs baseline (Pearson r = 0.75, *p* < 0.001), while the association was significant but negative when assessing the δThrombus vol and the δaortic lumen vol (Pearson r = −0.67, *p* < 0.001). Of note, volumetry was able to detect aneurysmal morphological changes in 94% of patients (48 out of 51), while the maximum aortic diameter found aortic enlargements in 67% of them (34 out of 51).

### 3.3. Associations of Hemostatic Factors with Image Parameters during AAA Progression

The analysis of image parameters showed that the majority of patients with non-surgical aneurysms (group 2) presented AAA progression in the short term, thus we decided to test whether the levels of the hemostatic makers TAT and D-dimer at baseline could predict AAA progression during 1-year independently of other confounding factors. By mix model regression analysis, we observed a significant direct association between the enlargement of AAA diameter, and the changes in thrombus volume and the aortic lumen volume, with the circulating TAT levels in univariate and multivariate analyses (*p* < 0.05, Table 3). 

**Figure 2:**
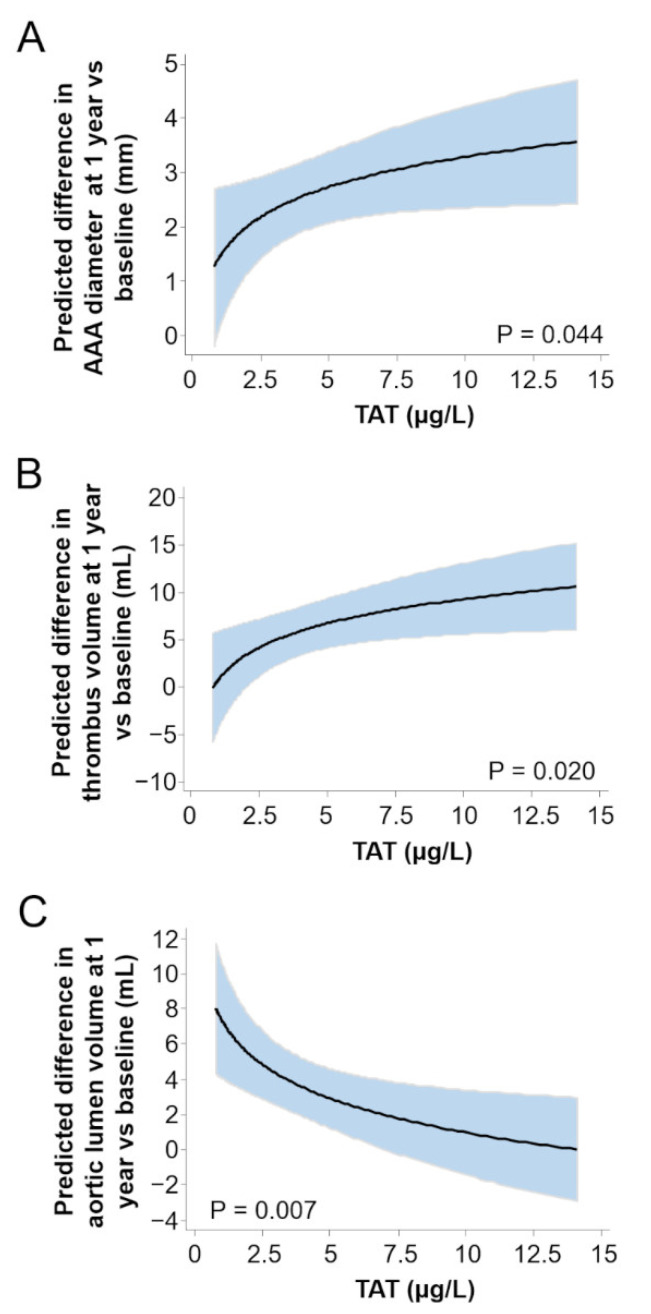
Hemostatic and image parameters correlate with AAA progression. (**A**–**C**) display the correlation between TAT levels and the differences in image parameters between 12 months and baseline for: (**A**) AAA maximum aortic diameter, (**B**) AAA thrombus volume and (**C**) aortic lumen volume in patients from group 2 (*n* = 51) adjusted for age, sex, smoking and dyslipidemia.

As illustrated in Figure 2A,B, high levels of TAT predicted AAA diameter and thrombus volume growth, while the aortic lumen remained similar (Figure 2C). As shown in Table 3, thrombus growth, and aortic lumen reduction correlated with increased baseline D-dimer levels.

### 3.4. Risk Prediction for Aneurysm Surgery in the Follow Up

**Figure 3:**
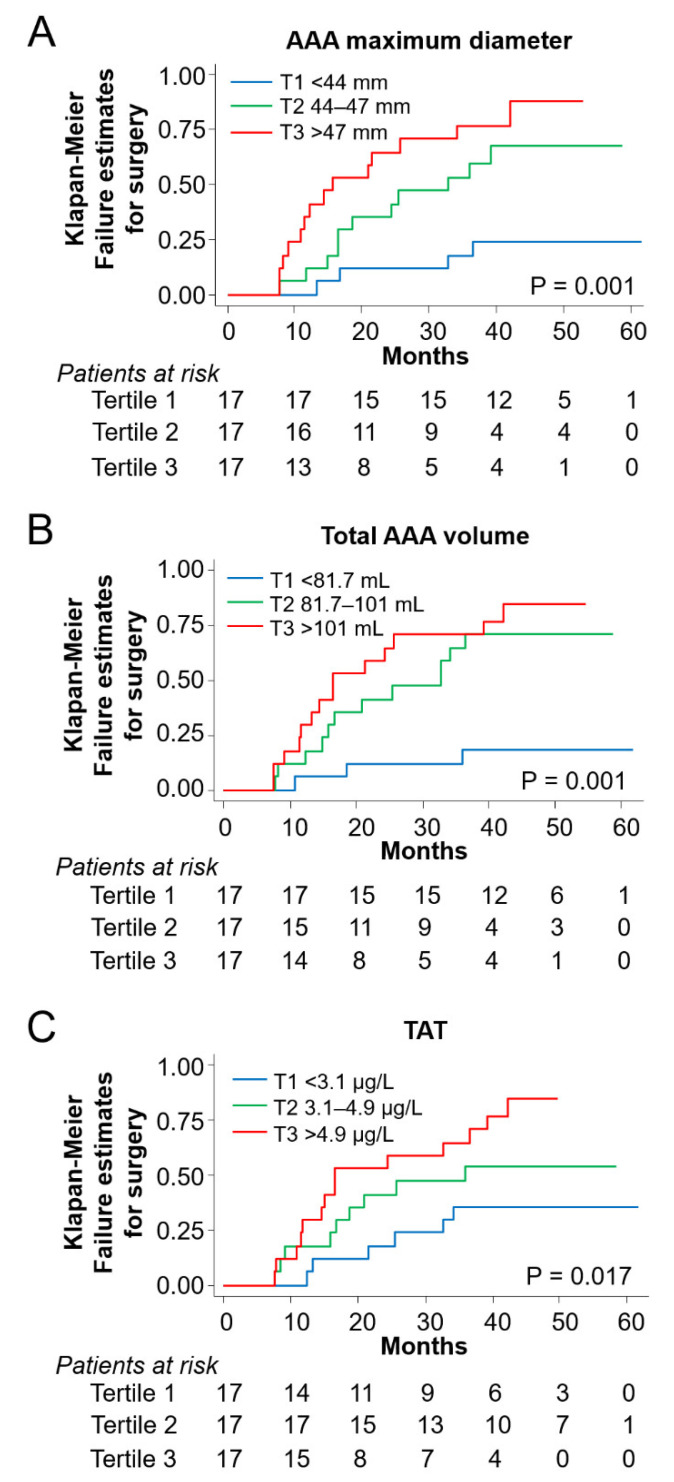
Hemostatic and image parameters correlate with worse outcome in patients with AAA. (**A**–**C**) Kaplan-Meier estimates for surgery in group 2 (*n* = 51, log-rank test) in relation to tertiles of: (**A**) AAA maximum aortic diameter, (**B**) total AAA volume, and (**C**) TAT levels.

Finally, to determine the value of image and hemostatic parameters at recruitment to predict the risk of aneurysm growth to the surgical limit (risk of surgery), patients in group 2 were follow-up for a mean of 4 years recording aortic surgery as outcome (*n* = 29, 57%). All baseline image parameters were associated to a higher probability of surgery before and after covariate adjustment (*p* ≤ 0.036, Table 4, Figure 3A,B). Regarding hemostatic proteins, TAT was associated with the outcome (Table 4), increasing 1.3-fold the risk of needing surgery in the follow-up for a doubling in TAT circulating concentration at baseline (*p* < 0.03 in all tested models, Table 4, Figure 3C). No association between the studied outcome and D-dimer was observed (SHR 1.24, 95%IC (0.92–1.68), *p* = 0.17).

## 4. Discussion

Predicting the progression of small aneurysms is a main challenge in AAA management and circulating biomarkers might provide an alternative for risk stratification in this pathology. In the current study we observed an increase in the hemostatic factors TAT and D-dimer in patients with AAA compared to non-AAA vascular subjects. We then explored, in the subgroup of patients with non-surgical AAAs, the association of those parameters with aneurysm diameter and volume variations during 1 year. Our results suggest that volumetry might be better suited to detect aneurysmal changes in the short-term as compared to the maximum diameter. We also observed an association between the procoagulant markers D-dimer and TAT with the progression of thrombus and aortic lumen volumes, and that of TAT also with the aortic diameter. Finally, all tested image parameters were associated with a higher probability of undergoing aortic surgery due to aneurysm diameter enlargement. Among the hemostatic variables only TAT was able to predict this outcome. Taken together, we provide herein valuable information supporting that the determination of some hemostatic parameters, and volumetry improve AAA patients’ characterization and outcome assessment, thus being clinically relevant.

In this study we have determined AAA size by two different techniques, maximum aortic diameter and volumetry, since no gold standard for its measurement has been yet defined. Indeed, despite being the most broadly used method to assess AAA growth, and the clear correlation between the maximum aortic diameter and the risk of AAA rupture, no consensus on its measurement protocol has been reached [3,4,5]. Moreover, by measuring the aortic diameter no reconstruction of the AAA sac three-dimensionally can be achieved [6]. Volumetry on the other hand, is broadly used to classify morphological changes in the aortic sac of patients undergoing endovascular surgery [6,7,9,16], and enables the determination of thrombus volume. The role of the aortic thrombus in AAA progression, however, is still controversial. Some authors postulate that the thrombus might protect the aorta from shear stress [17], while others propose that it might promote inflammation participating in aortic wall weakening [18]. According to previous reports [19], we observed herein a strong correlation between AAA volume changes and thrombus growth in the short-term, suggesting a possible role of the thrombus in AAA progression. Despite these data, using volumetry for the follow-up of small AAAs remains unclear. According to others [20], we observed a significant AAA enlargement over a year with both the aortic diameter and volumetry, although, the latter might be more appropriate to detect changes in the aneurysmal sac in the short-term. We also found a correlation between the aortic diameter and the AAA volume growth during 1 year, supporting, as already suggested by others [20], the benefit of combining the maximum diameter and the AAA volume to better define the aneurysm sac at baseline and during the follow-up. Finally, we observed that both techniques were able to predict the risk of undergoing surgery.

It is well known that the presence of AAA is associated to an increased procoagulant and profibrinolytic status [21] and thus we determined the levels of two hemostatic parameters for AAA progression assessment: TAT complexes and D-dimer as markers of thrombin generation and blood clotting, respectively. According to previous data [12,22,23], we observed a significant elevation of TAT complexes in patients with AAA compared with patients without AAAs supporting a hypercoagulable state associated to aneurysmal disease. Moreover, in the subgroup of patients with small aneurysms we found an association between TAT and the AAA diameter growth in the multivariate mix model regression analysis. Our findings are in line with those by Sundermann et al. showing that TAT was a strong predictor of AAA growth, assessed also by the aortic diameter, in a mean follow-up of 4.2 years [22], while we observed such an association already at 1 year, supporting a role of TAT as a marker of worse evolution earlier than probably predicted. Interestingly, TAT levels rendered an association with the expansion of thrombus volume, and a correlation with the reduction of the aortic lumen volume during one year. Additonally, TAT was the only hemostatic parameter associated to an increased probability of undergoing aortic surgery during the follow-up. Our data indicate that TAT might be a marker not only of the AAA diameter growth but also of the volume and hemostatic changes within the intraluminal thrombus, and suggest its possible utilization as a biomarker to assess the probability for mid-term surgery.

D-dimer is the most studied hemostatic marker in AAA and has been associated to aneurysm incidence and diagnosis [24]. In this line, we observed increased circulating D-dimer levels in patients with AAA compared with non-AAA subjects. Taking in consideration the nature of group 1, patients with atherosclerosis but without AAA, our results suggest a possible role of D-dimer for AAA diagnosis even alongside patients with atherosclerosis in other vascular beds, for instance PAD, also known to present elevated levels of this protein [25]. Additionally, D-dimer has been proposed as a marker of AAA growth, showing a strong correlation with aortic diameter progression [22,23,25,26], although we did not find such an association. It is worth considering, however, that those studies presented follow-up periods of at least 4-years, while our study was limited to 1-year and was performed in a smaller group of patients, therefore with a lower statistical power. Nonetheless, we did find associations between D-dimer and AAA thrombus volume growth, and aortic lumen volume reduction at 1-year corroborating previous data from other authors [27].

*Study limitations*: It is a prospective study with a limited number of patients. The follow-up, 1-year, enables the evaluation of short-term, rather than medium or long-term associations between image and hemostatic parameter. This could partially explain the lack of correlation between the maximum aortic diameter and D-dimer in our population, as previously reported by other authors with follow-up periods of at least 4 years [22,23,25,26]. In this regard, the presented data will need to be validated in larger patient cohorts at longer follow-up periods. No standardized methodology or guidelines are available for AAA volumetric assessment, hampering the comparison between different image techniques. Still, our semi-automatic measurement method, based on the 3mensio workstation, has been previously validated with good intra- and inter-observer variabilities for the volumetric determination of the aortic aneurysm [28]. Indeed, we report in here a difference in AAA volume of 9.4 ± 7.75 mL at 1 year, similar to the volume defined as significant for AAA progression (10 mL) in non-surgical AAA [8,29,30]. Despite offering the participation in the study equally to men and women, we have verified a significant difference according to the estimated proportion of men and women affected by AAA (4 male/1 female) [31]. Our non-AAA population (group 1) includes patients with atherosclerosis (coronary heart disease, cerebrovascular disease and/or peripheral artery disease) but without AAA, that might likely present increased levels of prothrombotic factors. Despite this fact, and the lack of specificity of D-dimer and TAT for AAA diagnosis and characterization, we observed increased levels of both coagulation markers in patients with AAA (groups 2 and 3) vs subjects without AAA. This might be related to the pathophysiological changes underlying AAA growth and intraluminal thrombus formation, that are closely related to deranged hemostasis. Aneurysm rupture occurs when the stress (force per unit area) on the aneurysm wall exceeds the wall force. Likewise, patients with ruptured/symptomatic AAA present increased peak wall stress (PWS) levels when compared with patients with AAA, or with subjects with electively repaired AAA [32,33,34]. Despite the importance of blood flow dynamics in AAA progression and rupture, we could not explore the possible relationship between flow parameters and hemostatic markers. In this regards, further studies will need to be performed to determine the relationship between blood flow, hemostatic markers and image parameter with AAA progression.

## 5. Conclusions

Our study suggests that volumetry might be more suited to detect aneurysmal changes in non-surgical AAAs in the short-terms as compared with the aortic diameter. However, the strong correlation between the aortic diameter growth and total volume expansion favors the combination of both to better characterize AAA size and progression. We also analyzed the value of hemostatic biomarkers to improve the classification and aneurysmal evolution of patients with AAA, reporting a correlation between D-dimer and TAT with both volumetry and aortic diameter changes over a year. Finally, baseline image parameters and circulating TAT levels were able to predict, in our cohort, the probability of AAA growth until surgery requirement in the follow-up, suggesting that patients with non-surgical AAA and elevated levels of TAT might benefit from a closer follow-up, for instance by increasing the frequency of image testing. Given the lack of effective treatments to prevent the formation or development of AAA, image and biological biomarkers such as volumetry and TAT might provide valuable tools to evaluate AAA size and worse evolution.

## Figures and Tables

**Table 1:** Clinical and demographical parameters of non-AAA vascular patients (group 1) and patients with AAA with aortic diameters between 40 and 50 mm (group 2) and >50 mm (group 3).

	Group 1 (*n* = 52)	Group 2 (*n* = 51)	Group 3 (*n* = 50)	*p* for Trend
***Demographical and clinical parameters***				
Sex (male, n, %)	40(77)	46(90)	50(100)	0.001
Age (years)	75(6)	72(8)	73(8)	0.161
BMI	27(4)	28(5)	28(4)	0.872
Smokers (n, %)	31(60)	45(88)	45(90)	<0.001
DM (n, %)	11(21)	12(24)	8(16)	0.630
Hypertension (n, %)	32(61)	34(67)	33(66)	0.839
Dyslipidemia (n, %)	38(73)	41(80)	38(76)	0.679
***Biochemical parameters***				
Total cholesterol (mg/mL)	180(37)	175(39)	175(44)	0.453
LDL (mg/dL)	109(33)	105(33)	107(38)	0.865
HDL (mg/dL)	48 ± 13	42 ± 10	40 ± 11	0.002
***Treatment (n, %)***				
Antiplatelets	31(60)	28(55)	41(82)	0.009
Anticoagulants	10(19)	11(22)	9(18)	0.900
ACE inhibitors	16(31)	14(28)	10(20)	0.450
ARA-2	12(23)	13(25)	11(22)	0.914
Calcium antagonists	8(15)	18(35)	11(22)	0.056
Vasodilators	3(6)	5(10)	6(12)	0.541
β-Blockers	10(19)	19(37)	20(40)	0.049
Statins	34(65)	41(80)	39(78)	0.171
Metformin	10(19)	10(20)	4(8)	0.190
***Previous history of CVD (n, %)***				
Coronary disease	11(21)	11(22)	15(30)	0.503
Stroke	5(10)	2(4)	3(6)	0.496
COPD	12(23)	11(22)	10(20)	0.931
CKD	2(4)	8(16)	4(8)	0.108
PAD	28(54)	9(18)	11(22)	<0.001
***Functional parameters***				
ABI	0.83(0.2)	0.92(0.2)	0.93(0.2)	0.016
***Hemostatic parameters***				
TAT (µg/L) *	2.2 (2.1)	4.1(3.7)	4.7(6.9)	<0.001
D-dimer (ng/mL) *	869 (1047)	1683(1958)	1832(1606)	<0.001

Mean (SD) is shown, except for * Log transformed variables presenting median (IQR, interquartile range). ABI: Ankle-brachial index, ACE: angiotensin-converting enzyme, ARA-2: angiotensin II receptor antagonist, CKD: chronic kidney disease, COPD: chronic obstructive pulmonary disease, CVD: Cardiovascular disease, PAD: Peripheral artery disease, LDL: low-density lipoprotein, HDL: high-density lipoprotein, eGFR: estimated-glomerular filtration rate, hs-CRP: high-sensitivity C reactive protein.

**Table 2:** Aneurysm growth in patients with AAAs of 40–50 mm (group 2, *n* = 51) at recruitment and 1 year after inclusion.

Image Parameter	Recruitment	1 Year	*p*	δ 1 Year- Recruitment
**Maximum aortic diameter (mm)**	45.04 (2.82)	47.63 (3.43)	<0.001	2.59 (2.53)
**AAA volume (mL)**	95.36 (24.25)	104.75 (28.75)	<0.001	9.40 (7.75)
**Aortic thrombus volume (mL)**	44.05 (22.87)	50.04 (27.59)	<0.001	5.98 (10.45)
**Aortic lumen volume (mL)**	51.30 (15.34)	54.72 (17.23)	0.001	3.41 (6.92)

Mean (SD) is shown. δ refers to the difference between the image parameters at 1 year and the values obtained at recruitment.

**Table 3:** Mix model regression analysis to assess the association of hemostatic parameters and AAA growth in patients with AAA between 40–50 mm (group 2, *n* = 51).

Image Parameter	TAT (µg/L) *			D-dimer (ng/mL) *		
	Mean Difference	95% CI	*p*	Mean Difference	95% CI	*p*
**AAA diameter (mm)**						
Unadjusted	0.56	0.01–1.11	**0.047**	0.23	−0.39–0.86	0.47
Adjusted	0.56	0.02–1.10	**0.044**			
**AAA volume (mL)**						
Unadjusted	0.65	−1.02–2.31	0.45	1.71	−0.09–3.51	0.06
Adjusted	0.65	−0.98–2.27	0.43	1.71	−0.05–3.46	0.06
**AAA Thrombus volume (mL)**						
Unadjusted	2.58	0.32–4.84	**0.025**	3.45	1.02–5.88	**0.005**
Adjusted	2.58	0.41–4.75	**0.020**	3.45	0.55–6.35	**0.020**
**Aortic lumen volume (mL)**						
Unadjusted	−1.93	−3.42–−0.44	**0.011**	−1.74	−3.41–−0.08	**0.040**
Adjusted	−1.93	−3.32–−0.54	**0.007**	−1.74	−3.32–−0.16	**0.030**

* Log2 transformed variables. The linear regression model includes: age, sex, smoking and dyslipidemia.

**Table 4:** Cox regression analyses (Fine-Gray model) to evaluate the associations between image parameters and TAT with the risk of undergoing surgery in patients with AAA between 40–50 mm (group 2, *n* = 51).

Models	AAA Diameter (mm)			AAA Volume (mL)			AAA Thrombus Volume (mL)			Aortic Lumen Volume (mL)			TAT (µg/L) *		
	SHR	95% CI	*p*	SHR	95% CI	*p*	SHR	95% CI	*p*	SHR	95% CI	*p*	SHR	95% CI	*p*
Unadjusted	1.35	1.11–1.64	**0.003**	1.03	1.02–1.04	**<0.001**	1.02	1.00–1.03	**0.015**	1.03	1.00–1.06	**0.023**	1.34	1.08–1.67	**0.007**
Model 1	1.45	1.20–1.75	**<0.001**	1.04	1.02–1.05	**<0.001**	1.02	1.01–1.04	**0.002**	1.03	1.00–1.06	**0.036**	1.36	1.05–1.76	**0.019**
Model 2	1.53	1.26–1.86	**<0.001**	1.04	1.02–1.05	**<0.001**	1.02	1.01–1.04	**0.003**	1.04	1.01–1.07	**0.012**	1.33	1.03–1.72	**0.029**
Model 3	1.45	1.20–1.75	**<0.001**	1.04	1.03–1.05	**<0.001**	1.02	1.00–1.04	**0.016**	1.03	1.00–1.06	**0.024**	1.38	1.07–1.79	**0.014**

* Log2 transformed variable. SHR: Sub-hazard ratio. Model 1: age, sex. Model 2: age, sex, dyslipidemia; Model 3: age, sex, smoking.

## Data Availability

The data that support the findings of this study are available from the corresponding author upon reasonable request.
